# A primary study of breeding system of *Ziziphus jujuba* var. *spinosa*

**DOI:** 10.1038/s41598-021-89696-1

**Published:** 2021-05-14

**Authors:** Feng Wang, Xiaohan Sun, Jibin Dong, Rong Cui, Xiao Liu, Xiangxiang Li, Hui Wang, Tongli He, Peiming Zheng, Renqing Wang

**Affiliations:** 1 Institute of Ecology and Biodiversity, School of Life Sciences, Shandong University, 72 Binhai Road, Qingdao, 266237 China; 2 Shandong Provincial Engineering and Technology Research Center for Vegetation Ecology, Shandong University, Qingdao, China; 3 Qingdao Forest Ecology Research Station of National Forestry and Grassland Administration, Shandong University, Qingdao, China

**Keywords:** Ecology, Evolution, Plant sciences

## Abstract

*Ziziphus jujuba* var. *spinosa* has been used as a windbreak and for soil conservation and water retention. Previous studies focused on pharmacological effects and extraction of chemical components in this species, and very few explored the breeding system. The present study combined the analysis of floral morphology, behavior of flower visitors, and artificial pollination to reveal reproductive characteristics of the species. Its flowers are characterized by dichogamy, herkogamy, and stamen movement, which are evolutionary adaptations to its breeding system. There were more than 40 species of visiting insects, mainly Hymenoptera and Diptera, and the characteristics of dichogamous and herkogamous flower adapted to the visiting insects. The breeding system is outcrossing, partially self-compatible, and demand for pollinators. The fruit setting rate after natural pollination was 2%. Geitonogamy and xenogamy did not significantly increase the fruit setting rate, indicating that the low fruit setting rate was not due to pollen limitation by likely caused by resource limitation or fruit consumption. The fruit setting rate of zero in emasculated and in naturally and hand self-pollinated individuals suggested the absence of apomixis and spontaneous self-pollination. The above results can be utilized in studies on evolution and cultivation of *Z*. *jujuba* var. *spinosa.*

## Introduction

Breeding system in plants is an essential part of their life cycle and an important factor in their evolution^1^. About 80% of angiosperms contain bisexual flowers, including hermaphroditism, monoecy, andromonoecy, gynomonoecy, and trimonoecy^2^. Among those, hermaphrodites account for 70% of angiosperms and are generally considered to be ancestral types of angiosperms^3^. Understanding the reproductive characteristics of hermaphrodites will thus help us to interpret plant evolution. Studies have linked interference between sexual functions to hermaphroditism, and the theory of gender interference helps to explain the evolutionary adaptation of floral characteristics^4^. The movement of stamens and pistils provides additional evidence for the adaptive significance and evolutionary ecology of flowers^5,6^. Different pollination methods were used to deal with a series of plants in order to realize the discussion of their reproductive ecological characteristics in previous studies^7^. Some hermaphroditic flowering plants have a temporal separation of male and female functions, which is traditionally considered an effective mechanism to prevent self-pollination in flowers ^8^.

The evolution of floral characteristics is influenced by pollinator choice and co-evolution with pollinators, and is the main driving force for angiosperm speciation^9–11^. Attracting more pollinators to participate in pollen transfer and thereby improve the success rate of pollination is an important driving force of flower evolution^8^. The evolution of floral characteristics is not only related to pollinators, but also influenced by environmental factors, genetic linkage of different genes, and metabolism^12–14^. Flower color affects the behavior of pollinators and improves pollination efficiency^15^. Flowering phenology may be an important factor in maintaining the diversity of plant communities^13^, and it is also the result of selection pressure, which can reflect the suitability of plants^16^.

Although self-pollination may be an important component of the population mating system, previous research showed that insects play a role in promoting cross-pollination^17^. The destruction of habitats by human activities and climate change has dramatically reduced biodiversity^18^ and disrupted the plant–pollinator relationship^19,20^. The functional diversity of pollination networks helps to maintain the diversity of plant communities^21^. About 87.5% of flowering plants rely on animals to deliver pollen^22^. Globally, the value of pollinators for crop production in 2005 was estimated at 153 billion euros per year^23^. Governments, media, and scientists all over the world have raised concerns about the reduction in pollinator numbers and the loss of pollination services^24^.

In the natural state, plants may self-pollinate, cross-pollinate with the help of insects and other external factors, or both. Different pollination methods have different effects on fruit and offspring quality^25^. Many studies on reproductive ecology of plants implemented artificial control of pollination. Generally, self-pollination may result in less seed setting and seed yield^26^ and the seed setting is significantly improved by cross-pollination^7,27^. Artificial pollination ensures that plants receive enough pollen, which is then used to study pollen restrictions^28,29^. For example, female and male reproductive success of female, male, and hermaphrodite individuals in the subdioecious shrub *Eurya japonica* (Theaceae) was studied using artificial pollination^30,31^. Thus, artificial pollination is suitable for studies on reproductive ecology.

As a soil and water conservation species, *Ziziphus jujuba* var. *spinosa* acts as a windbreak, protects from wind-blown sand, and reduces soil erosion. The previous studies on this species were mainly focused on its pharmacological effects, such as extraction of chemical components^32–35^, and on the effects of water, nutrition components, and arbuscular mycorrhizal fungi on its physiology and ecology^36,37^. *Z. jujuba* var. *spinosa* is an excellent species of soil and water conservation in China. Its performance of forest and slope protection has obvious advantages in improving the ecological environment. It is also a new generation of wild fruit resources with great development potential, and has high economic and medical value. It is of great ecological significance to improve the germination rate and seedling survival rate of wild *Z. jujuba* var. *spinosa* seeds for the conservation and restoration of vegetation and the reduction of soil erosion. There is few researches focusing on the breeding system of *Z. jujuba* va*r. spinosa.* Some researchers studied the pollen of *Z*. *jujuba*, and found that there were significant differences in pollen germination rate in different stages. The results of in vitro culture were more accurate^69,70^.

The aim of the present study was to reveal the characteristics of pollination biological and reproductive system of *Z. jujuba* var. *spinosa* by examining floral morphology and conducting insect trapping and artificial pollination. The following specific topics were discussed: (1) the flower morphology adapting to its breeding system in evolution; (2) the relationship between the flowers and flower-visiting insects (species, quantity and behavior); (3) breeding system of *Z. jujuba* var. *spinosa* (self-crossing or cross-breeding). The results presented herein would provide information that can be used to study the evolution and cultivation of *Z*. *jujuba* var. *spinosa*.

## Result

### Floral features

**Figure 1:**
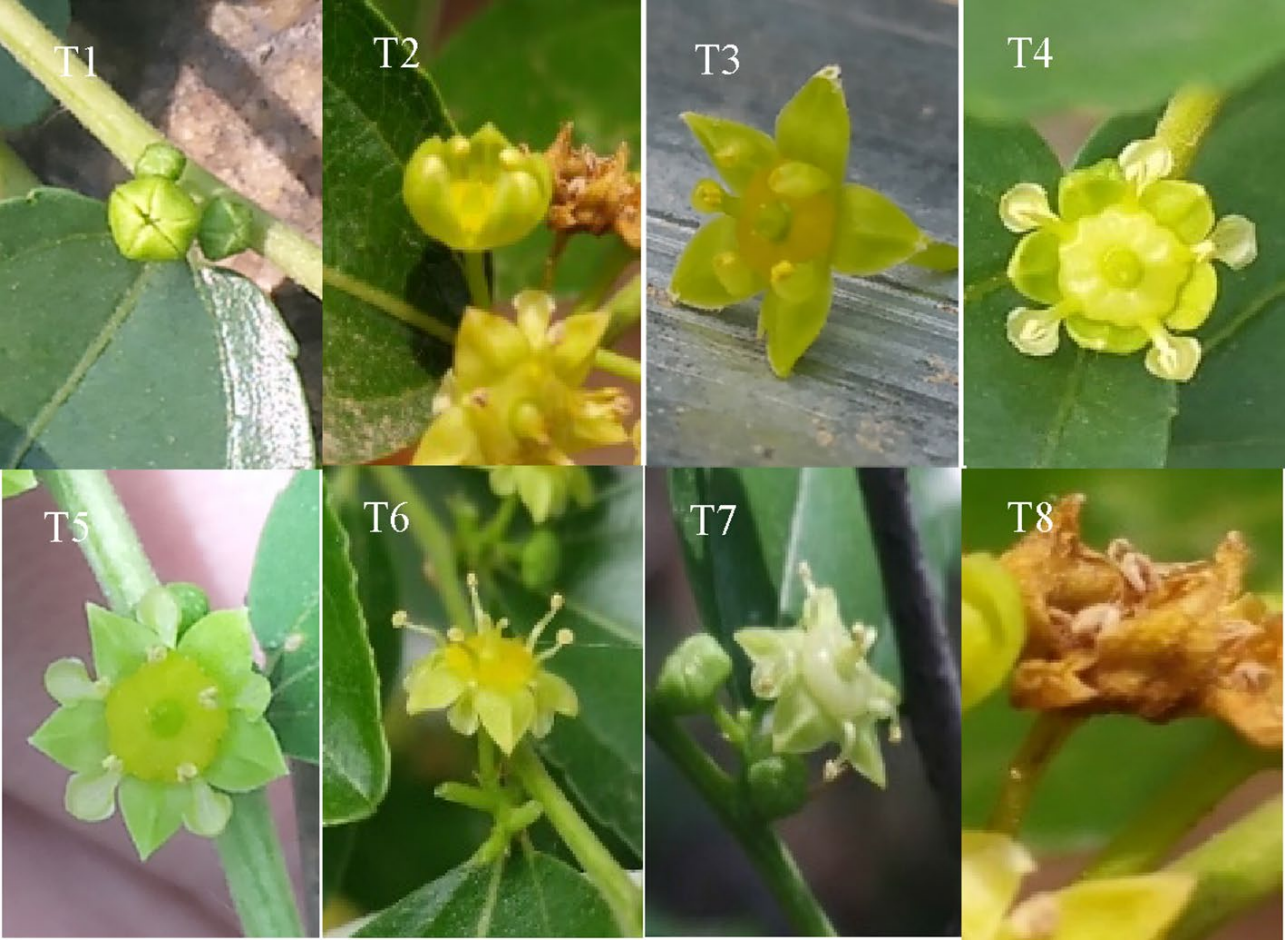
Single flower opening stages. T1-bud opening stage (bud dehiscence, showing no internal structure of flower), T2- initial opening stage (sepals initially open, showing the internal structure of flowers), T3-sepal flattening stage (sepals spreading horizontally, petals still enveloping stamens), T4- petal and stamen separation stage (petals open, separate from stamens), T5- petal flattening stage (petals horizontally distributed between sepals), T6- stamen flattening stage (the stamens bend to the horizontal direction, when the stigma is already bifurcated), T7-stamen drooping stage (stamen bending downward), T8- wilting stage.

**Figure 2:**
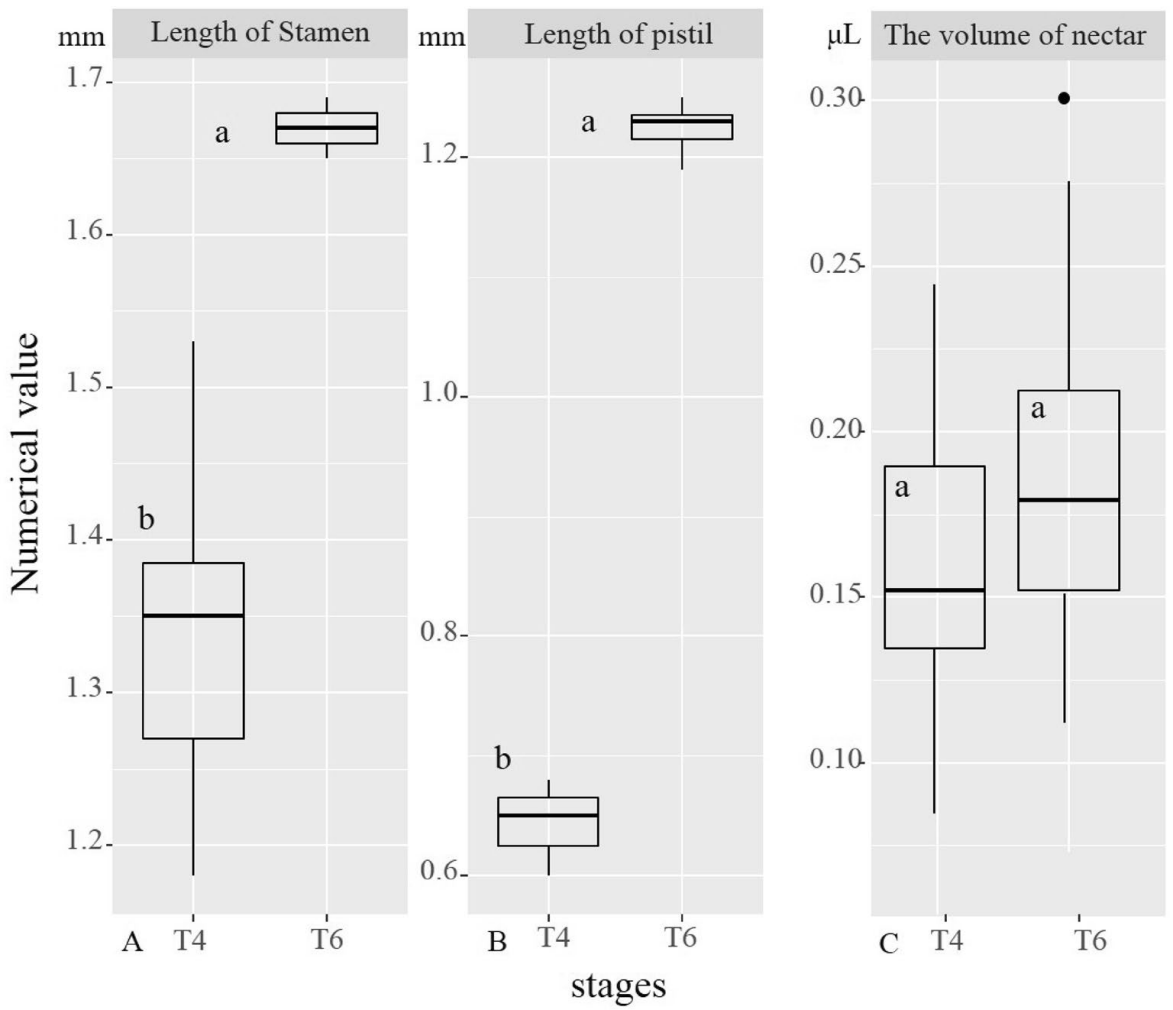
The length of stamen (**A**), length of pistil (**B**) and volume of nectar (**C**) at T4 and T6 periods were plotted. The lines in the box represent the median value of the data (n = 30). Different letters indicate a significant difference based on a generalised linear mixed model (GLMM). A shows that the length of stamens in T4 period is 1.34 ± 0.11, and the length of stamens in T6 period is 1.67 ± 0.01. *P*= 0.002 < 0.05. There were significant differences between the two groups. B shows that the length of pistil in the T4 period is 0.64 ± 0.03, and the length of pistil in the T6 period is 1.22 ± 0.02. *P*= 1.28e−14 < 0.001. There are very significant differences between the two groups. C shows that the nectar volume in the T4 period is 0.14 ± 0.04, and the nectar volume in the T6 period is 0.17 ± 0.06.  *P*= 0.178 > 0.05. There were no clear significant differences between the two groups.

Eight stages were identified from bud to senescence stage of a single *Z*. *jujuba* var. *spinosa* flower (Fig. 1): T1-bud opening stage (bud dehiscence, no internal structure of the flower), T2-initial opening stage (sepals initially open, internal structure of the flower visible), T3-sepal flattening stage (sepals spreading horizontally, petals still enveloping stamens), T4-petal and stamen separation stage (petals opened, separated from stamens), T5-petal flattening stage (petals horizontally distributed between sepals), T6-stamen flattening stage (the stamens bend to the horizontal direction, when the stigma is already bifurcated), T7 -stamen drooping stage (stamen bending downward),T8-wilting stage. T1–T5 stages occurred on the first day of flowering; T6 stage was reached the second day of flowering; and T7 stage followed one day later. The flowers are hermaphroditic. The flowers are hermaphroditic. Stamens and pistils were elongated in shape during the flowering (Fig. 2A,B), and the stigma was bifurcated. Stamens moved slowly, from straight position to horizontal, and finally bending downward (Table 1); the color of petals and the pistil changed during the flowering period (Table 1).

**Table 1:** The functional floral morphology of *Z. jujuba var. spinosa.*

Items of observation			Results of observation
Flower organs wilting order			Petal → Stamen
	Color changes		Aqua (No obvious changes)
Petal development	State changes		Wrapping → Separating → Flattening → Drooping
	Color changes		Canary → Brown
Stamens development	Filament changes		Short → Long Curve → Upright → Curve
	Distance from anthers to stigma		Short → Long
	Mode of anther dehiscence		Longitudinal dehiscence
	Number		4,5,6
Pistil development	Stigma	Color	Aqua → Brown
		Shape	Cone → Bifurcate → Long
	Ovary	Color	Yellow → Ivory → Cyan(Fertilization) → Red(Withered)

### Nectar volume

Nectar was secreted on the first and second day of flowering, and nectar secretion was measured in the T4 and T6 stages (Fig. 2C). The nectar volume in T4 and T6 was 0.14 ± 0.04 and 0.17 ± 0.06, respectively. There was no significant difference in nectar production between T4 and T6 stages (P > 0.05).

### Pollen germination rate

**Figure 3:**
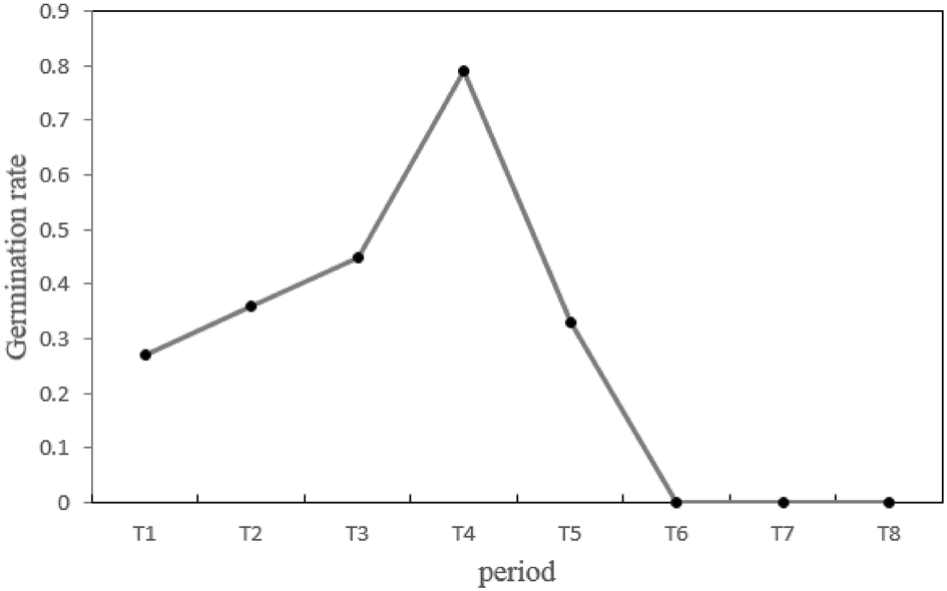
Pollen germination rate of Z. *jujuba* var. *spinosa* at different flowering stages.

The number of pollen grains in the petal flattening stage (T6) and later was very limited and difficult to collect. Therefore, the pollen for pollen germination rate experiment was collected from flowers between the bud opening stage (T1) and the petal flattening stage (T5) (Figs. 1 and 3). The pollen germination rate gradually increased from the bud opening stage, peaked at the petal and stamen separation stage (T4), and declined thereafter. Overall, pollen activity was the highest at the petal and stamen separation stage (T4).

### Flower visitors and their visiting behavior

**Figure 4:**
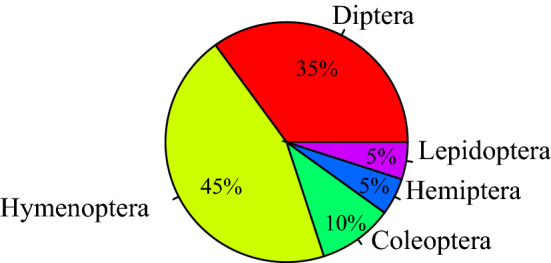
species of flower visiting insects.

About 40 species of insects, belonging to five orders, visited the flowers. The largest order was Hymenoptera, accounting for 45% of total insect visitors, followed by Diptera (35%), Coleoptera (10%), and Hemiptera (5%) and Lepidoptera (5%) (Fig. 4).

**Figure 5:**
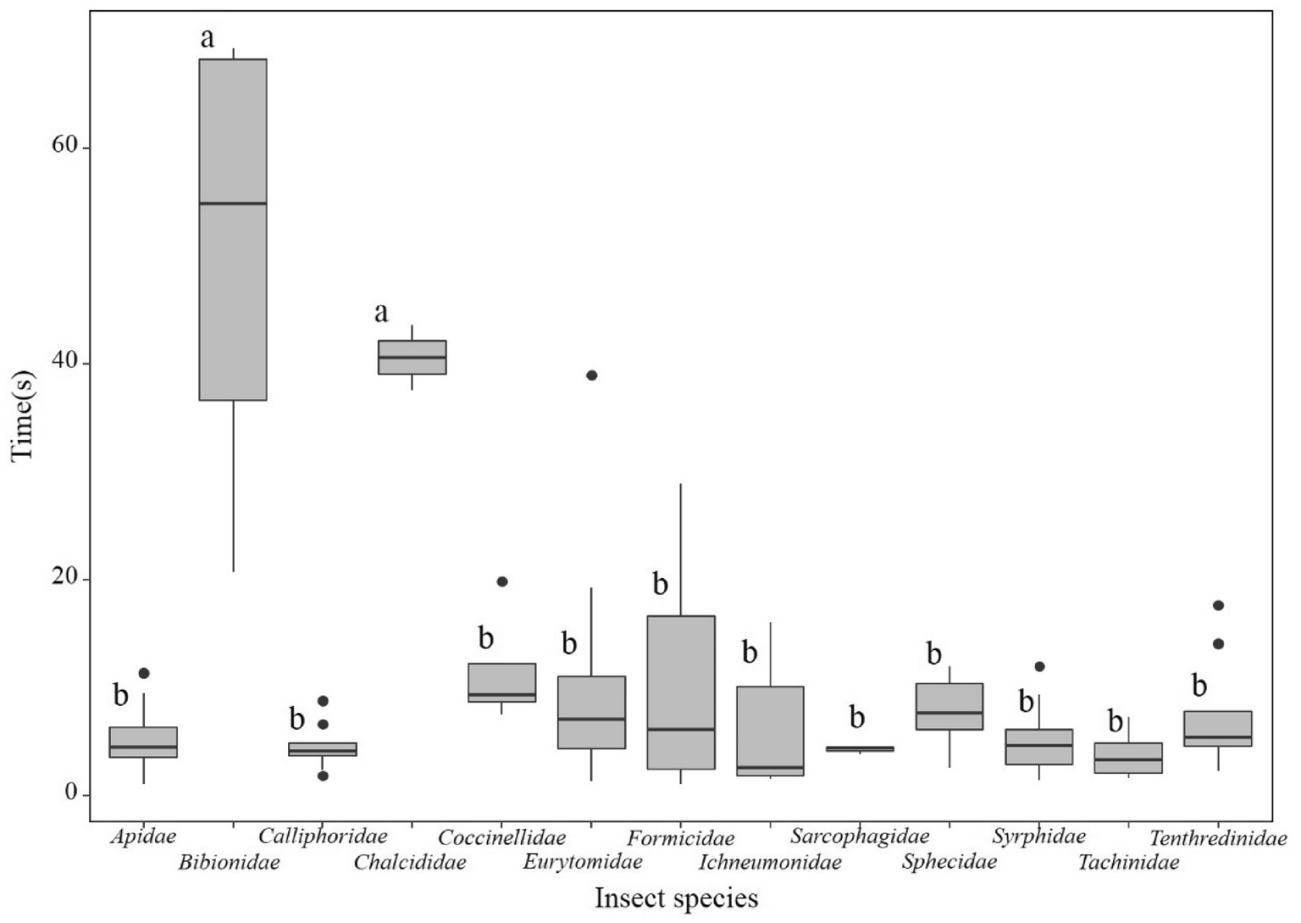
Observed visiting time of different families. The lines in the box represent the median value of the data. Different letters indicate significant differences in the results of multiple comparisons in which family-wise errors were adjusted using Tukey’s method. P < 0.01.

According to our investigation, Apidae and Syrphidae are the main flower visiting insect families. Moreover, the operating time of the visiting insects on the flower differed between insect families. The visitation time of Bibionidae and Chalcididae was significantly longer than that of the other families (*P* < 0.01; Fig. 5). Differences were also observed in the visitation time among Bibionidae individuals (Fig. 5).

**Figure 6:**
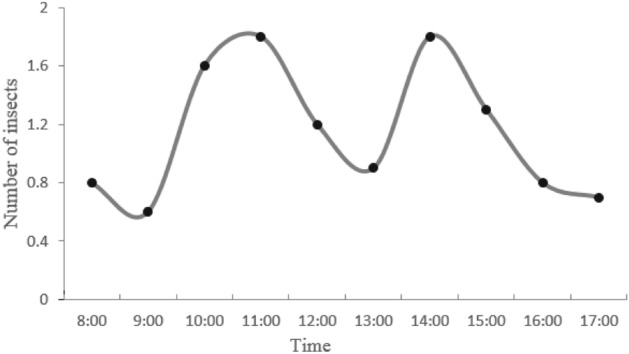
Average frequency of insect visiting flowers (between 8:00–17:00).

Figure 6 shows the daily visitation frequency of the insects from 8:00 to 17:00. The visitors were much more active at 11:00 and 14:00, and much less dynamic in the mornings (8:00–9:00), at dusk (16:00–17:00), and at noon (12:00–13:00).

### Pollen-ovule ratio (P/O) and Outcrossing index (OCI)

*Ziziphus jujuba* var. *spinosa* has two ovules^38^. The average amount of pollen in a single flower was 7800 ± 300 grains (*n* = 30). The pollen-ovule ratio (P/O) was 3900 ± 150, indicating that the breeding system is xenogamy^39^. The OCI was 4 and calculated by summation of the following values: (1) flower diameter was 5.13 ± 0.22 mm (*n* = 30), and assigned a value of 2; (2) the stamen was mature before the pistil, and therefore its value was 1; (3) the pistil and stamen were spatially separated, hence the value of 1^40^. According to Dafni standard, the breeding system is mainly outcrossing, partially self-compatible, and demands pollinators.

### Artificial pollination experiment

**Figure 7:**
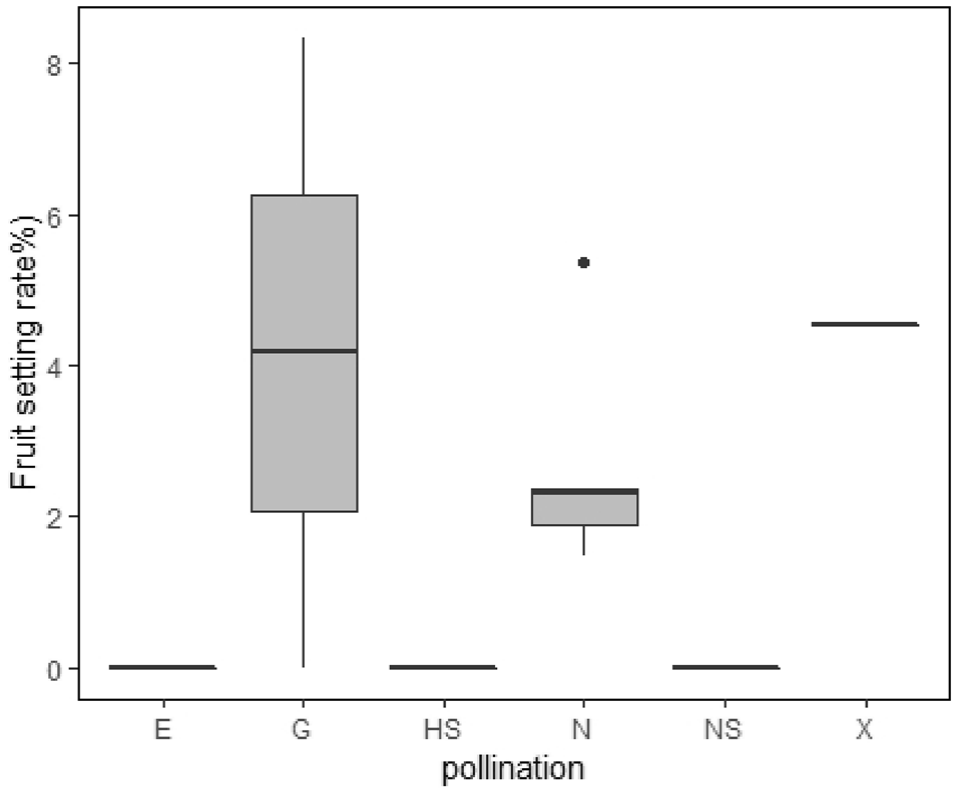
Fruit setting rate of artificial pollination experiment.

Figure 7 shows the fruit setting rate of various artificial pollination treatments. The fruit setting rate of emasculated, hand self-pollinated, and naturally self-pollinated flowers was 0% (Fig. 7). The fruit setting rate of geitonogamy and xenogamy was about 4%, which was slightly higher than that of natural pollination (2%); the difference was not significant.

The upper and lower hinges of the box indicate 75 and 25th percentiles of the data, respectively. E, Emasculated; G, Geitonogamy; HS, Hand self-pollination; N, Natural pollination; NS, Natural self-pollination; X, Xenogamy. There was no significant difference between G, N and X.

## Discussion

### Adaptive evolution of floral morphology

Self-pollination in hermaphrodites has advantages, but inbreeding also has a detrimental effect. During evolution, plants tend to cross-pollinate and evolve toward avoiding self-pollination^41^, such as herkogamy, dichogamy, and hermaphroditism^5^. The obvious way to promote outcrossing is by preventing self-pollination. A decline in self-pollination has been observed in a large number of plants, and there is evidence that the process of self-pollination is genetically controlled^42^. Studies have shown that dichogamy is a common but neglected feature of outcrossing in angiosperms. Dichogamy can reduce self-interference and thus reduce selfing^43^. The observations of the flowering period in *Z*. *jujuba* var. *spinosa* revealed the characteristics of herkogamy and protandry (Figs. 1 and 2), both of which are mechanisms that tend to avoid self-pollination.

Four types of stamen movement has been reported in previous studies, and they include stimulated, simultaneous and slow, quick and explosive, and cascade^44,45^. In this study, we found that stamen movement in *Z. jujuba* var. *spinosa*. belongs to simultaneous and slow type. Stamen movement of some plants can promote self-pollination, increase the probability of pollination, and facilitate reproduction^46,47^. However, the slow movement of stamens does not promote self-pollination, but supports cross-pollination. Although herkogamy can reduce the possibility of selfing, the success of pollination is reduced because of the difference between pollinator contact sites on the anthers and stigmas, resulting in wasted resources^48^.

The pollen germination rate confirms the protandrous nature of the *Z*. *jujuba* var. *spinosa* reproductive system (Fig. 3). The stamens are scattered from the bud opening stage (T1) to the petal flattening stage (T5). At this time, the pistils are immature, and the stigmas are not receptive (Fig. 1). Similar pistil and stamen characteristics were reported for some species in Labiatae^49^. This mechanism can effectively avoid self-pollination and increase the rate of outcrossing^48,50^.

The nectar volumes in T4 (petal and stamen separation stage) and T6 (stamen flattening stage) were similar (Fig. 2C), indicating that pollinator attraction by nectar is always imparted during stamen (T4) and stigma maturation (T6). This further favors the outcrossing.

### Characteristics of flower visiting insects

The sexual system of plants is fundamentally related to the pollination biology of plants^51^. Pollinators play important roles in plant reproduction^52^. Biological pollination is considered a key factor in biodiversity of natural populations^53^, and plays an indispensable role in evolution. The productivity of crops can be improved by increasing species diversity and quantity of local wild flowers around farmlands^54^.

Hymenoptera and Diptera are the dominant visitors of *Z. jujuba* var. *spinosa* (Fig. 4). These orders include important flower visitors and pollinators of many plant species and in various habitats^55^. Although the pollination efficiency of non-bee insects is not as high as that of bees, they visit flowers more often and play an important role in global crop production^56^. In fact, the species and number of pollinators are related to the pollination background and weather conditions—more kinds of insects are active on sunny days, and the opposite is true on cloudy days^57–59^.

Additionally, the frequency of insect flower visitations is related to flowers morphology and environmental factors, such as temperature or light intensity. In the morning and afternoon, the frequency of insect visits was increased (Fig. 6). The decrease in flower visitation frequency at noon may be due to high temperature and strong solar radiation^60^, which is not conducive to vigorous activities of the insects. At this time, some insects hide under the leaves to avoid strong solar radiation. The flower visiting behavior of insects and the characteristics of flower adapt to each other^61^, which is helpful for *Z. jujuba* var. *spinosa* to avoid inbreeding and increase the probability of outcrossing.

### Breeding characteristics of *Z. jujuba* var. *spinosa*

The study of plant reproductive biology helps us to understand the reproductive strategies in different species, which will ensure successful reproduction as well as facilitate population survival and community evolution^62^. The breeding system of *Z. jujuba* var. *spinosa* was analyzed and determined through estimating the pollen-to-ovule ratio (P/O) and the outcrossing index (OCI), and by artificial pollination. The pollen ovule ratio (P/O) of a single flower was 3900 ± 150, which belonged to specific outcrossing^39^. The OCI value of 4 was indicative of the primarily outcrossing breeding system, and partial self-cross compatibility and need for pollinators^40^. The two indexes thus indicate outcrossing as the breeding system in *Z. jujuba* var. *spinosa*.

The breeding system of this species was further investigated through artificial pollination. The fruit setting rate after natural pollination was only 2%, which may have been caused by certain limiting factors. Namely, geitonogamy and xenogamy did not significantly increase the fruit setting rate, indicating that pollen number was not a limiting factor. Thus, the low fruit setting might be due to resource limitation or fruit feeding^63^. In this experiment, the fruit setting rate of Emasculated is 0, which indicates that there is no apomixis. The fruit set rate of Natural-self-pollination and Hand-self-pollination are all 0, indicating that there is no self-pollination. Certain species cannot self-pollinate even if they are inbred^64^. Inbreeding decline affects the evolution of mating system and the persistence of small mutation load in a population^65^. Declining tendency for inbreeding and preference for outcrossing is present in many species^66,67^. The floral characteristics of *Z. jujuba* var. *spinosa* reflect the adaptation to its breeding system. Dichogamy can ensure that self-pollination is avoided, but it can't prevent Geitonogamy. Because of the decrease in fitness caused by excessive far crossing, part of self compatibility (i.e. Geitonogamy) becomes the most suitable form of mating^68^. It can improve genetic diversity and promote plant evolution while ensuring the success of reproduction.

## Material and methods

### Study species and site

*Ziziphus jujuba* var. *spinosa* is a hermaphroditic shrub or small tree in the Rhamnaceae. It is used as an excellent soil and water conservation species in China, and it is an important nectar and entomophilous plant.

This study was carried out on Qiangu Mountain, Qingdao City, Shandong Province, China (lat.36°29′53.33″N, long.120°43′44.31″E). The annual average temperature in the region is 12.1 °C, the maximum temperature is 38.6 °C, and the minimum temperature is − 18.6 °C. The annual average precipitation is 708.9 mm, 65% of which occur in summer.

The study was conducted at three sites, each with more than 10 individuals of *Z*. *jujuba* var. *spinosa*. Additional dominant trees in each site included *Pinus densiflora* Siebold & Zucc., *Robinia pseudoacacia* L., *Quercus acutissima* Carruth., and *Vitex negundo* L. var. *heterophylla* (Franch.) Rehder.

### Floral morphology

Three individuals of *Z*. *jujuba* var. *spinosa* were randomly selected at each site, and three inflorescences were randomly chosen in each individual (about 40 flowers develop on each branch) to investigate the flowering process and floral morphology (Fig. 1). The number of flowers, flower shape, size, and color at different flowering stages were recorded (Table 1).

### Nectar volume

Flowers were bagged with paper pollination bags to exclude pollinators before evaluation. According to the single flower opening stages of *Z. jujuba* var. s*pinosa*, there was less nectar from T1 stage to T3 stage (Fig. 1). On the first day, the attraction of flowers to visiting insects mainly began in T4 stage, and on the second day, it mainly began in T6 stage. Nectar volume per flower was evaluated at petal and stamen separation stage (T4) or stamen flattening stage (T6) after anthesis using a 1 μL microcapillary tube.

### Pollen germination rate

Pollen germination rate was determined by in vitro culture method. Six flowers were collected at each flower developmental stage and fresh pollen was shaken onto a slide coated with a thin layer of MS medium. The total number of pollen was ≥ 600. The slides were placed in a petri dish with two layers of wet filter paper to keep the culture environment moist. The slides were cultured in a dark incubator at 26 °C, and pollen germination rate was examined after 24 h under an optical microscope (CX31RTSF; Olympus, Tokyo, Japan). The germination rate was calculated using the following formula:

$${R}_{PG}(\%)=\frac{{n}_{l}}{{N}_{t}}*100$$

where ${R}_{PG}$ is pollen germination rate (%), n_l_ is the number of pollen grains with tube length longer than pollen diameter, N_t_ is the number of pollen observed.

### Flower visitors and their visiting behavior

From June 4 to June 11, 2019, the flower visiting insects were investigated hourly on marked inflorescences or individuals from 7:00–18:00 after the plants entered the full bloom period. The species and their behavior were observed and recorded. Flower visitation frequency was measured by stopwatch and camera, and the following three aspects were recorded: (1) the time needed for insects to operate flowers (such as drilling into the corolla) and to ingest nectar; (2) the time needed for insects to fly from one flower to the next; and (3) the pattern of insect movement among flowers.

### Pollen-ovule ratio (P/O) and outcrossing index (OCI)

Ten mature but indehiscent anthers were collected from three individuals (30 anthers in total) and placed in three centrifuge tubes, each containing 10 mL 0.1% hydrochloric acid solution. Pollen grains in each tube were incubated in a water bath with shaking for 4 h at 55 °C. The suspension was removed from the bath and immediately transferred onto a slide, and the number of grains was recorded. P/O was determined as the ratio of pollen grain number per flower (the number of grains per anther multiplied by the number of anthers) to the number of ovules.

The Dafni’s OCI^40^ is calculated at the species level based on floral morphological characteristics and used to predict the mating system. The value of OCI is obtained by adding the scores of each judgment condition. The OCI of *Z*. *jujuba* var. *spinosa* was calculated based on inflorescence diameter, flower size, and flowering behavior according to Dafni.

### Artificial pollination experiment

From May to July, 2018, five mother trees were randomly selected at every site. Two flower branches 20–40 cm long were randomly selected for each mother tree, and flower buds on each branch were marked and counted. To prevent natural insect pollination, the selected branches were covered with nylon bags. The following treatments were conducted:

- Control 1: Natural pollination, no emasculation, no bagging (N);
- Control 2: Natural self-pollination, no emasculation, bagging (NS);
- Treated 1: Geitonogamy, emasculation, and bagging (G);
- Treated 2: Hand self-pollination, no emasculation, bagging (HS);
- Treated 3: Xenogamy, emasculation, and bagging (x);
- Treated 4: Emasculation, bagging (to test whether self-pollination occurred at budding stage) (E).

During the experiment, three male parents were selected for pollen selection, and the suitable flower stage was chosen for pollination. Pollen was collected with a brush and transferred onto the stigma several times in succession.

## Data Availability

All data generated or analysed during this study are included in this published article (and its Supplementary Information files).
